# Diagnostic Ability of Radiofrequency Ultrasound in Parkinson’s Disease Compared to Conventional Transcranial Sonography and Magnetic Resonance Imaging

**DOI:** 10.3390/diagnostics10100778

**Published:** 2020-10-02

**Authors:** Mindaugas Baranauskas, Rytis Jurkonis, Arūnas Lukoševičius, Vaidas Matijošaitis, Rymantė Gleiznienė, Daiva Rastenytė

**Affiliations:** 1 Biomedical Engineering Institute, Kaunas University of Technology, K. Baršausko g. 59-454, LT-44029 Kaunas, Lithuania; rytis.jurkonis@ktu.lt (R.J.); arunas.lukosevicius@ktu.lt (A.L.); 2 Department of Neurology, Medical Academy, Lithuanian University of Health Sciences, A. Mickevičiaus g. 9, LT-44307 Kaunas, Lithuania; vaidas.matijosaitis@lsmuni.lt; 3 Department of Radiology, Medical Academy, Lithuanian University of Health Sciences, A. Mickevičiaus g. 9, LT-44307 Kaunas, Lithuania; rymante.gleizniene@lsmuni.lt

**Keywords:** Parkinson’s disease, brain tissue micromovements, displacement waveform, radiofrequency ultrasound, transcranial sonography

## Abstract

We aimed to estimate tissue displacements’ parameters in midbrain using ultrasound radiofrequency (RF) signals and to compare diagnostic ability of this RF transcranial sonography (TCS)-based dynamic features of disease affected tissues with conventional TCS (cTCS) and magnetic resonance imaging (MRI) while differentiating patients with Parkinson’s disease (PD) from healthy controls (HC). US tissue displacement waveform parametrization by RF TCS for endogenous brain tissue motion, standard neurological examination, cTCS and MRI data collection were performed for 20 PD patients and for 20 age- and sex-matched HC in a prospective manner. Three logistic regression models were constructed, and receiver operating characteristic (ROC) curve analyses were applied. The model constructed of RF TCS-based brain tissue displacement parameters—frequency of high-end spectra peak and root mean square—revealed presumably increased anisotropy in the midbrain and demonstrated rather good diagnostic ability in the PD evaluation, although it was not superior to that of the cTCS or MRI. Future studies are needed in order to establish the true place of RF TCS detected tissue displacement parameters for the evaluation of pathologically affected brain tissue.

## 1. Introduction

Parkinson’s disease (PD) is the second most common progressive neurodegenerative disease whose main neuropathological feature is the loss of dopaminergic neurons of the substantia nigra (SN) located in the midbrain [1,2] and the deposition of α-synuclein in neurons [3]. Despite huge achievements in contemporary neurology and neuroimaging, a definitive diagnostic test for PD is not yet available [4]. Positron emission tomography and single-photon emission computed tomography are currently the most common functional radionuclide diagnostic methods used in degenerative extrapyramidal disorders; however, their application in clinical practice remains limited due to high price, the relatively short half-life of the radioisotopes, radiation exposure to patients, and the discrepant data on their reliability in differential diagnosis [4,5,6]. Advanced magnetic resonance imaging (MRI) techniques and post-processing procedures are also being extensively explored to enhance diagnostic accuracy for PD [7,8], nevertheless, these novel techniques are also rather costly and not easily accessible.

Transcranial sonography (TCS) is a relatively new method for assessment of the deep brain structures, including midbrain and SN [9,10,11]. Hyperechogenicity of SN in a cross-sectional B-mode (Brightness or Grayscale mode) image of the midbrain is treated as a main PD biomarker [4]. Nevertheless, TCS needs to be performed by an experienced examiner to be reliable and support the diagnosis for the patient. Therefore, additive techniques are acknowledgeable in order to improve diagnostic reliability of TCS, especially if they are inexpensive, non-invasive, safe and easily accessible.

TCS modification opens up the possibility to track the brain tissue displacements at the scale of micrometers caused by the endogenous quasi-periodic pulsatility in the intracranial basin. Endogenous characteristics of brain tissue displacements are supposedly related with the biomechanical features of pulsating tissue and are likely affected by evolving pathology and correlated with tissue stiffness [12]. These brain tissue displacements can be evaluated by detailed analysis of raw ultrasound (US) radiofrequency (RF) signal changes of the same tissue region over time [13]. We developed such an approach further [14] and in our recent study [15] we demonstrated that evaluation of complex interactions between the set of RF TCS-based brain tissue displacement parameters allows us to discern the medial temporal lobe of an Alzheimer’s disease patient from that of a healthy control (HC) subject with excellent diagnostic ability.

The aim of this study was to estimate tissue displacement parameters in midbrain using backscattered US RF signals and to compare the diagnostic ability of RF US-based dynamic features of disease affected tissues with conventional TCS (cTCS) and MRI while differentiating patients with PD from HC.

## 2. Materials and Methods

The design and consent procedures of this exploratory study were approved by the Ethics Committee for Biomedical Research at the Lithuanian University of Health Sciences (decision on 19 December 2017, No. BE-2-728), Kaunas, Lithuania. All participants gave signed informed consent prior to inclusion in the study. All procedures were in accordance with the Declaration of Helsinki.

### 2.1. Patients and Control Group

Twenty PD patients were prospectively selected from both outpatient and inpatient units of the Department of Neurology at the Hospital of Lithuanian University of Health Sciences Kaunas Clinics (Kaunas, Lithuania) from March 2018 until March 2019.

Neurologists who consulted the patients for a movement disorder performed an initial selection of potential study subjects. Patients were included in the study if they: (1) were diagnosed with idiopathic or familial PD, which was based on the United Kingdom Brain Bank criteria [16]; (2) provided written consent; (3) had satisfactory acoustic window properties on at least one side for TCS; (4) were 18 years or older; (5) were eligible for MRI. Patients were excluded from the study if they: (1) had an uncertain diagnosis; (2) had a major somatic disease (decompensated heart failure, terminal renal or hepatic dysfunction, active cancer, had diagnosed hemodynamically significant intracranial/extracranial artery stenosis or thrombosis); (3) had a severe mental disorder (psychotic type, severe depression); (4) had a prominent neurological deficit (severe visual disturbance, aphasia, severe paresis, ataxia).

Twenty age- and sex-matched HC subjects were recruited from healthy relatives of the patients, provided they did not have any major somatic illness as listed above, cognitive impairment or any structurally abnormal findings in brain MRI and were not under investigation or treatment for any neurodegenerative diseases. All participants were Caucasians and most of them were Lithuanians.

All participants completed a questionnaire on general demographic information and risk factors. Education was evaluated by the duration of formal education in years. Family history was considered positive if there was at least one known PD case among first- or second-degree relatives. The Motor section of the Unified Parkinson’s Disease Rating Scale (UPDRS) [17] was applied while assessing functionality of the patient.

### 2.2. Radiofrequency Transcranial Sonography (RF TCS)

RF TCS imaging for endogenous brain tissue motion data collection was performed using a research-dedicated ultrasound scanner, Ultrasonix Sonix Touch (Analogic Ultrasound, Richmond, BC, Canada, 2013), equipped with a phased array sector probe (SA 4–2, 64 acoustic elements). The main parameters of ultrasonic scanning and RF signal digitization were as follows: sampling frequency fs = 40 MHz, analog-to-digital converter resolution 16 bits, number of post-beam formed scanning lines—131, angle of phased array transducer sector—60°, scanning depth—11 cm, frequency of ultrasound waves 2.5 MHz, frame rate—45 Hz, transmit focal single depth—7 cm. The sequences for recording B-mode scans (259 frames in total) and raw B scan forming RF signals were acquired and stored for off-line analysis.

**Figure 1:**
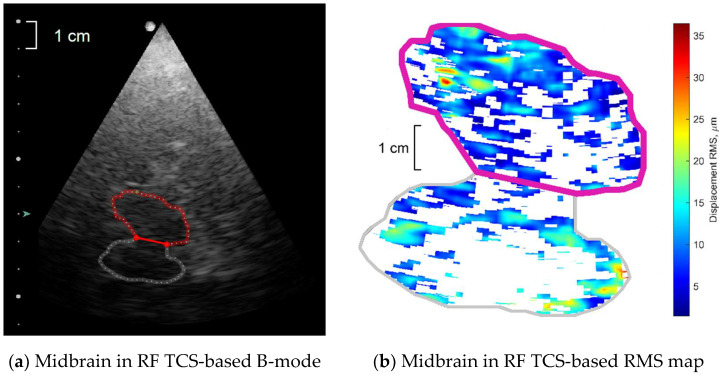

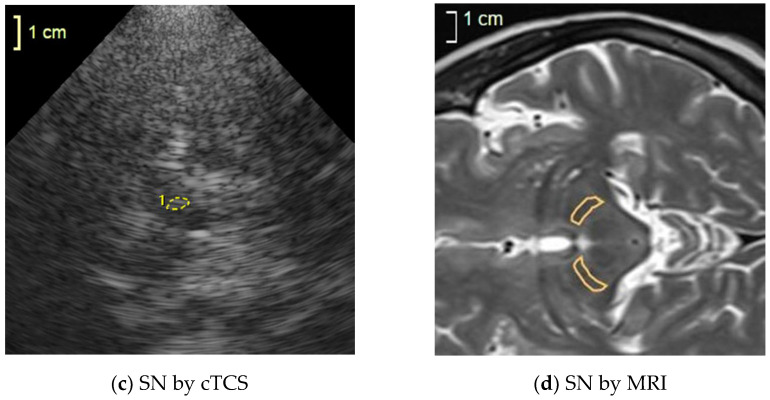
Regions of interest (ROI) examples in axial scanning plane images co-registered by different methods for the same healthy participant: (**a**) half of the midbrain in radiofrequency (RF) transcranial sonography (TCS) data-based B-mode image, (**b**) analyzed half of the midbrain and not-analyzed half of the midbrain mapped as root mean square (RMS) of confident displacements detected from RF TCS signal sequences; (**c**) substantia nigra (SN) in conventional TCS (cTCS) image of midbrain (**d**) SN by magnetic resonance imaging (MRI) of midbrain, T2W/TSE/2 mm sequences. Contours of ROI depicted in colorful lines (see online version). In sonography examples, the ultrasound transducer was on the left side of the head or at the top of images.

All US scans were performed by an experienced neurosonologist (Vaidas Matijošaitis) with 13 years of experience in intracranial vascular and structural ultrasound. All study subjects were asked to stay calm and relaxed in a supine position for 5 min and remain motionless and speechless during the examination. First scans were made in a midbrain (axial) scanning plane in B-mode through a right temporal bone acoustic window. The position of the transducer was fixed by closing the dedicated spherical bearing of the bracket during insonation of the midbrain. After recording the sequence of B-mode scan images, the butterfly shape midbrain was marked as a region of interest (ROI) for further investigation (see Figure 1a), and 6-s length US RF signal sequences were stored in the computer memory. The investigator had no contact with the patient, US transducer or the patient’s bed during US RF signal recording. All procedures described above were repeated while making scans through a left temporal bone window. Thus, two ROIs—one from the left and another from the right side of the head—were investigated separately for each study subject.

The classical 1D cross-correlation [18,19,20] was used to obtain spatial point displacements in ROI along the scanning line from the acquired US RF signals. The calculation options used at this step were the same as described in our earlier article [14]. The displacement signal processing was the same as in our recent study [15]: the coordinate system was changed into Lagrangian [21], the median for each scanning frame’s line was subtracted separately and high-pass filtered with cutoff at 0.75 Hz and only confidently repeatable moving spatial points were selected for further analysis.

The waveforms of displacement signals were quantified using four groups of the displacement parameters for each individual confident spatial point:

1. Amplitude parameters:
  - Root mean square (RMS)—represents displacement intensity over an entire recorded time (6-s). RMS of each individual confident spatial point depicted in Figure 1b.
  - Peak-to-peak amplitude of a mean repeated movement; this parameter was called “pulse amplitude” by Kucewicz et al. [19].
2. Strain parameter—Lagrangian strain as module of the derivative of amplitudes of a mean repeated movement calculated along the ultrasound scanning line’s direction.
3. Morphology parameter—frequency of high-end spectra peak (shortly–FreqHP) was calculated from the entire displacement signal length (6-s). FreqHP was estimated at the peak of the power spectra observed in the frequency range from 1.5 × FreqD to 22.5 Hz, where FreqD is the dominant frequency in displacement low-end spectra (from 0.67 to 2.00 Hz interval) supposedly caused by heart beats.
4. Energy parameter—relative energy within the 4–6 Hz frequency band normalized to total energy of displacement signal. This parameter was introduced to control the Parkinsonian 4–6 Hz rest tremor [22,23]. Distribution of this energy parameter was evaluated only by its statistical mode.

The distributions of remaining parameters—amplitude, strain and morphology group parameters of all displacement signals of individual points—were quantified by their minimal and maximal values, median, first (Q1) and third quartiles (Q3), interquartile range (IQR) and the most frequent value (statistical mode); in addition, amplitude group parameters were also evaluated by ex-Gaussian estimates *μ*, *σ*, *τ*, also ex-Gaussian distribution mean and standard deviation (SD) using the DISTRIB toolbox for MATLAB [24]; as strain has similar to exponential distribution, only the ex-Gaussian distribution τ estimate was added from the ex-Gaussian estimates. Most of above-mentioned parameters and their distributions were described in more a detailed manner in our previous study [15].

### 2.3. Conventional TCS (cTCS)

All PD patients and HC subjects underwent cTCS with a commercially available ultrasound system, Voluson 730 Expert BT08 (General Electric (GE) Healthcare, Zipf, Austria). The ultrasound system was equipped with an electronic sectored PA2-5P phased array transducer, which has a working frequency range of 1.3 to 4.0 MHz, 128 piezoelectric elements, a modifiable 90-degree visual angle. B-mode images have 8-bit depth (i.e., 256 gray tones), a dynamic range of 180 dB and a maximum depth of 30 cm. The system comes installed with algorithms to reduce noise and artifacts.

TCS imaging was done using the PA2-5P/NEURO transducer mode. Scans were done in B-mode without any color coding or Doppler mode. Amplitude gain was changed manually, which was done by observing the live TCS image. The brightest point on the screen was used as a reference point for amplitude gain. Regulator sliders of time gain compensation were used and placed in a semicircle with convexity to the right so that the ROI, the midbrain along with its structures—SN, the red nucleus, raphe nuclei—could be seen the brightest.

All subjects were supine in a darkened room, scans were performed by placing the transducer at the level of the eyebrows with the front part pointed upwards, i.e., to the side of face at the pre-auricular temporal bone area. Scans were performed in two standard planes: (a) the mesencephalic or the midbrain plane, and (b) the diencephalic plane or the third ventricle plane. In the mesencephalic plane, the area of the SN projection was measured (in cm^2^) at the level of the transducer (see Figure 1c). Measurement of the height of the third ventricle in the segment neighboring brain stem was also performed.

All participants had quantitative measurements of the size of the third ventricle and the SN performed twice on each side by the same neurosonologist who performed RF TCS. The penetration depth of TCS was usually 16.8 cm, while the zoom was 1.6 times to measure the SN and third ventricle dimensions. The normative threshold values of the SN area calculated in our laboratory for Voluson 730 Expert BT08 were < 0.20 cm^2^ (mean + 1 SD) and < 0.26 cm^2^ (mean + 2 SD) [25]. For the third ventricle diameter, the normative value was <1.0 cm (mean + 2 SD) [26].

### 2.4. MRI Acquisition

All MRI scans were obtained using a 1.5 T Siemens MAGNETOM Avanto (Erlangen, Germany) scanner within 2 to 4 weeks from the examination by TCS. The imaging protocol included axial T2W/TSE/2 mm (TR 4740 ms, TE 3.37 ms, TI 1100 ms, flip angle 120), T1W/mpr/p2/iso (TR 3000 ms, TE 89 ms), T2W/fl2d/hemo (TR800 ms, TE 26 ms, flip angle 20), coronal T2W/TSE (TR 5000 ms, TE 93 ms, flip angle 150), DW/ADC (TR 3000 ms, TE 89 ms), axial and coronal T2W/FLAIR (TR 9000 ms, TE 98 ms, TI 2500 ms, flip angle 150) and sagittal T2W/spc2d/iso (TR 3200 ms, TE 379 ms) sequences of the entire brain. No contrast media were injected. No hardware or software upgrades of the MRI scanner were done during the study period.

All sequences (T2W/FLAIR, T2W, T2W/fl2d/hemo, DW/ADC) were used to eliminate intra- and extra-axial lesions (tumors, vascular pathology, etc.). For the measurements of the SN, T2W/TSE/2 mm axial images of midbrain were used in all PD patients and in HC. In the axial plane, SN is as crescent-shaped region (see Figure 1d); therefore, two quantitative measurements—ventral and dorsal—were taken. The ventral measurement was a maximum width of the visible SN in the cerebral peduncle near the interpeduncular cistern and 1.5 mm proximal from the inner corner of the midbrain. The dorsal measurement was a maximum width of the visible SN in the cerebral peduncle near the peripontine cistern and 2.0 mm distal from the inner corner of the midbrain. The area of SN was evaluated in the same sequence and scan level using the JiveX Diagnostics Advanced 5.2.0.3 system (VISUS Health IT GmbH, Bochum, Germany) measurement tool (Poligon statistic measurement).

### 2.5. Statistical Analysis

RF TCS, cTCS and MRI data were made quantitative in this study. This allowed us to provide detailed statistical analysis.

Normality of data distributions were examined using the Shapiro–Wilk test. Since majority of RF TCS-based parameter estimates were not normally distributed, they were compared between HC subjects and patients with PD using the non-parametric Mann–Whitney U test; remaining variables were compared between groups by a parametric Student t test for independent samples. Variables that achieved a *p* value of less than 0.20 were examined with multivariate analysis using logistic regression (LR) for three methods—RF TCS, cTCS and MRI—based measures separately. Forward and backward stepwise LR was applied to assess the predictive characteristics of the amplitude, strain and morphology (FreqHP) groups of parameters between PD patients and control subjects, with age (for all three methods based quantitative measures) and relative energy in the 4–6 Hz frequency band (only for RF TCS-based quantitative measures) included as a covariate.

Receiver operating characteristic (ROC) analysis was used to evaluate the performance of the diagnostic ability of the analyzed parameter estimates and—more generally—to evaluate the accuracy of the LR model that classified subjects into sick or healthy. An optimal diagnostic cut-off point was determined via the Youden index. ROC curves were analyzed to define a cut-off value for the highest sensitivity and specificity of predicted probability of LR models and the RF US parameter estimates, which achieved p ≤ 0.05 using a Mann–Whitney U test.

The significance level α was set at 0.05. Statistical analysis was performed using IBM SPSS Statistics 22.0 (IBM Corp., Armonk, NY, USA) software.

## 3. Results

### 3.1. Demographic Data and Single TCS and MRI Quantitative Measures

Initially, among PD patients 12 (60%) were men, 11 (50%) of HC subjects were men, and, thus, groups were similar regarding sex (χ^2^ test, *p* = 1.00, *p* > 0.05). Age and education of the initial sample did not differ statistically between the HC and PD groups (see Table 1: all subjects).

Once the US RF data were processed, six subjects from the PD patient group and one subject from the HC group were excluded due to an insufficient number of repeated waveforms in displacement signals (see Table 1: subjects with repeatable waveforms in RF TCS recordings). Therefore, US RF data of 19 control subjects (the ROIs of both sides for 10 subjects, only right ROI for three subjects, only left ROI for six subjects—29 ROIs in total) and of 14 PD patients (the ROIs of both sides for six patients, only right ROI for four patients, only left ROI for four patients—20 ROIs in total) were used for the final analyses. Ten persons (52.6%) of the control group were men, and nine (64.2%) were men in the PD group; however, groups did not differ by sex (χ^2^ test, *p* = 0.72). Age and education of the remaining subjects did not differ statistically between the HC and PD groups (see Table 1) too.

The morphology parameter FreqHP Q3 was the only parameter from RF US-based parameters that significantly differed statistically between HC (Me = 2.37, Q1 = 1.95, Q3 = 2.62, IQR = 0.68) and PD groups (Me = 2.71, Q1 = 2.20, Q3 = 3.30, IQR = 1.10) according to the Mann–Whitney U test (*p* = 0.018). The area under a curve (AUC) of ROC for FreqHP Q3 was 70.0% (95% confidence interval: 55.1–84.9%); at cut-off of 2.62 Hz, sensitivity was 65.0%, specificity was 75.9%. Besides, there was tendency that maximal FreqHP value (*p* = 0.076), RMS ex-Gaussian SD (*p* = 0.080) and RMS IQR (*p* = 0.091) was higher in PD group too. The dominant frequency (FreqD) of the low-end spectrum peak (expected heart rate) did not differ between groups (Mann–Whitney *p* = 0.282). Relative energy in 4–6 Hz frequency band did not differ between HC and PD groups too (Mann–Whitney *p* = 0.222).

The SN area had remarkable discrepancy between quantitative measures by MRI and by cTCS, these measures had moderate negative correlation (Pearson *r* = −0.498, *p* < 0.001). Scores on Motor section of UPDRS did not correlate with size of the SN area neither by MRI (*p* = 0.752), nor by cTCS measures (*p* = 0.487).

### 3.2. Models of Logistic Regression (LR) Analysis

To assess the predictive power of multiple parameters together for the likelihood that the subject had PD, forward and backward stepwise LR was applied.

From all 40 RF TCS-based parameter estimates, nine had a Mann–Whitney *p* value of less than 0.20, and thus initially we tried to pass them into LR; however, both forward and backward stepwise LR analysis produced models without RF TCS-based variables (i.e., both models only contained an intercept).

**Figure 2:**
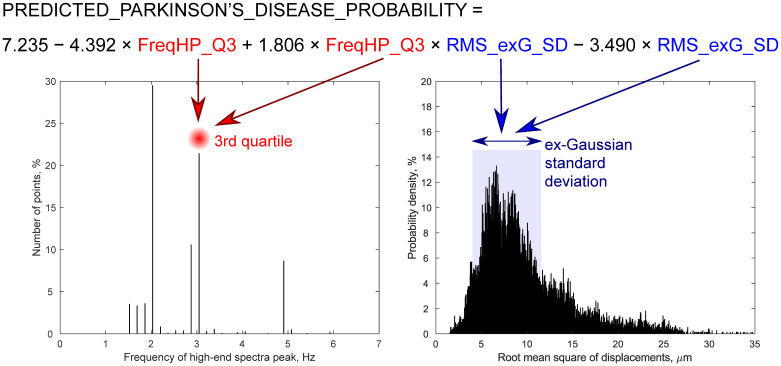
Visualization of radiofrequency ultrasound-based displacement parameter estimates that were included in the first logistic regression model. Sub-images represent distributions of displacement parameters of confidently repeatable moving individual points, from midbrain: (**a**) frequency of high-end spectra peak (FreqHP) and (**b**) root mean square (RMS). FreqHP_Q3—FreqHP third quartile, Hz; RMS_exG_SD—RMS standard deviation of ex-Gaussian distribution, µm.

Then, we evaluated multiple sets of RF TCS-based variables (four variables having a Mann–Whitney *p* value of less than 0.10) with their interactions manually. Only variables not exceeding 0.5 value of Spearman correlation were included into the one set for the LR model. According to ROC analysis, the most optimal LR model was with two single variables (FreqHP Q3 and RMS ex-Gaussian SD) and their interaction (see RF TCS 1st Model in Table 2 and Figure 2). After adding age covariate into this LR model, age became a statistically significant variable; however, two RF TCS-based components (one single variable and interaction) remained statistically significant (see RF TCS 2nd Model in Table 2). Relative energy in the 4–6 Hz frequency band was not a statistically significant variable after adding it as a covariate; however, then only one RF TCS-based component (interaction) remained at the threshold of statistical significance (see RF TCS 3rd Model in Table 2). All these three models were statistically significant (*p* < 0.005) and had a good classification ability (AUC > 80%) according to the ROC analysis (see Table 3 for more details).

The predicted probability of these RF TCS data-based LR models did correlate with the size of the SN area as measured by cTCS (from Spearman *rho* = 0.459 and *p* = 0.002 for RF TCS 1st Model to Spearman *rho* = 0.531 and *p* < 0.001 for RF TCS 2nd Model) and MRI (from Spearman *rho* = −0.347 and *p* = 0.015 for RF TCS 1st Model to Spearman *rho* = −0.517 and *p* < 0.001 for RF TCS 3rd Model). Predicted probability of these LR models did not correlate on the Scores on Motor section of UPDRS (*p* > 0.05).

Meanwhile cTCS and MRI quantitative data based LR models had an excellent classification ability (AUC > 90%) even with a single variable—size of SN area, independent of age (see Table 2 and Table 3).

## 4. Discussion

We aimed to assess the diagnostic ability of endogenous brain tissue displacement parameters in a midbrain using US RF signals and compare it with a diagnostic ability of the measurements of the SN area by cTCS and by MRI for the differentiation of patients with PD from HC subjects. The model constructed of RF TCS-based tissue displacement parameters—FreqHP and RMS—demonstrated quite good diagnostic ability in the evaluation of the midbrain.

The interaction between one morphology parameter (FreqHP) distribution estimate and one amplitude parameter (RMS) distribution estimate had the highest influence in the RF US-based model (RF TCS 1st Model). FreqHP reflects the distribution of frequency values by finding a peak with the maximum power within high-end spectra for every spatial point separately. Since this displacement waveform morphology parameter estimate Q3 was the only one from all US RF-based variables differing between HC subjects and PD patients (only Q3 of FreqHP became higher in PD patients), we can hypothesize that the registered micromovements in most parts of the midbrain did not significantly change with the disease, and the changes occurred only in part of the midbrain which had a relatively sharper micromovements pattern. The same FreqHP parameter-captured disease affected other brain structures in our previous study [15]. There, a set of brain tissue displacement signal parameters (FreqHP maximum, mode and IQR, and strain) worked together well while differentiating the medial temporal lobe of an Alzheimer’s disease patient from that of an HC subject with an excellent diagnostic ability [15]. The observed tendency that PD has wider RMS dispersion (as evaluated by ex-Gaussian SD and IQR) also suggest increased anisotropy in the midbrain.

The obvious difference in the results using the method under investigation could be caused by differences of the constitutive properties of tissue between various regions of the brain, between white matter and gray matter [27,28,29], by the specificities of the underlying neurodegenerative process, by cardiovascular pulsatility [30] and cerebral autoregulation [31] and by the method itself.

The brainstem, including the midbrain, is globally stiffer than the cerebral hemispheres [27], and is anisotropic [32,33] because the largest part of brainstem consists of ascending and descending axons forming major tracts, and white matter tissue is found to be stiffer than gray matter [29]. In the case of PD, a neurodegenerative process in the midbrain affects mainly dopaminergic neurons of the compact part of the SN which forms a less-stiff gray matter nucleus. The SN is a very small part (small percentage) of the entire midbrain and, in contrast to Alzheimer’s disease, does not result in apparent atrophy or other changes on conventional neuroimaging modalities such as computed tomography or MRI [34]. Deformation-based morphometry and independent component analysis, however, identified PD-specific atrophy in the midbrain, basal ganglia, basal forebrain, medial temporal lobe and discrete cortical regions [35]. Since the SN area is taking a relatively small part in the midbrain scanning plane, therefore, observed changes by our RF TCS-based micromovements tracking method are relatively subtle; however, they are still captured by this method.

Besides, according to our data, the midbrain is located about 1.5 times deeper than the medial temporal lobe assessed in our previous study. Therefore, the US scanning inter-line gaps are increased from 0.54 to 0.73 mm, although this loss of resolution is hardly influencing our results. However, transtemporal scanning perpendicular to the cranio-caudal axis of the pulsating brain [36,37] might limit the possibility of detecting the endogenous deformations more significantly.

Despite all the conditions mentioned above, diagnostic ability findings for the RF TCS of the midbrain (when age included as covariate, sensitivity is 80.0%, specificity is 86.2%) appear to be similar to that recently published for the cTCS of the SN (sensitivity: 85%, specificity: 89% respectively) [38], as well as to our previously published results (sensitivity: 90%, specificity: 82.4%, respectively [25].

To our knowledge, our study is the first one providing quantitative measures obtained by RF TCS (with high amount of various parameter estimates), cTCS and MRI methods simultaneously. In our present study, the predicted probability of PD was excellent (ROC AUC ≥ 90%) with a sensitivity of 90–95% and a specificity of 96.6–100% for both structural TCS and MRI measurements of the SN area, i.e., a bit higher compared to that reported by other researchers. Conventional T1- and T2-weighted MRI measurements of the SN area were criticized for poor delineation of the SN and we found just two published studies looking at the differences of the measurements of the SN area between PD patients and HC subjects with conflicting results [39,40]. In a study by Minati et al. [39], the SN area of the PD patients was significantly smaller compared to that of HC subjects while no difference was found in a study by Oikawa et al [40]. Recently the SN pars compacta T1w/T2w ratio value on 3T MRI was proposed as a novel, parsimonious in vivo biomarker for the PD [41].

It should be noted, however, that our current study was not designed as a diagnostic accuracy study, the number of participants was quite small and investigators were not blinded to the clinical status of the subject, and this might have had some influence on the results. A high percentage of PD patients’ data were excluded due to non-repeated movement of the brain tissue.

We believe that an assessment of endogenous brain tissue displacement parameters in order to detect pathologic changes specific for PD in a midbrain using backscattered US RF signals still has a future, as it has potential for the improvement in respect of sonography scanning planes and transcranial acoustic windows, enabling detection of stronger displacement of midbrain endogenous motion along the cranio-caudal axis. Furthermore, more parameters, for instance, relative strain could be informative in the semi-longitudinal section of a midbrain. Future studies could validate our results using larger samples of participants to try to achieve a better balance between temporal and spatial resolution with the RF TCS method, which could ideally open the possibility to locate the small SN structure and simultaneously capture its micromovements to try to better integrate data while evaluating SN or midbrain properties by simultaneously using several methods such as RF TCS and cTCS.

## 5. Conclusions

The model constructed of RF TCS-based brain tissue displacement parameters—FreqHP and RMS—revealed, presumably increased anisotropy in the midbrain and demonstrated rather good diagnostic ability in the PD evaluation, although it was not superior to that of the structural cTCS or MRI. Future studies are needed in order to establish a true place of US RF signal-based displacement parameters for the detection of pathologically affected brain tissue.

## Figures and Tables

**Table 1:** Demographic characteristics, conventional transcranial sonography and magnetic resonance imaging quantitative measures. Statistically significant differences according to the Student t test marked in bold.

Variable	Mean ± Standard Deviation		*p* Value
	HC	PD	
**All subjects**			
Age, years	68.5 ± 6.8	64.1 ± 10.1	0.17
Education, years	15.2 ± 3.2	14.7 ± 2.9	0.61
Motor section of UPDRS	–	33.2 ± 12.9	–
**Subjects with repeatable waveforms in RF TCS recordings**			
Age, years	68.1 ± 6.8	62.9 ± 10.7	0.123
Education, years	15.0 ± 3.2	14.9 ± 3.2	0.99
Motor section of UPDRS	–	31.6 ± 10.7	–
SN dorsal width by MRI, mm	4.29 ± 1.29	3.08 ± 1.77	**0.007**
SN ventral width by MRI, mm	5.35 ± 1.31	4.05 ± 1.44	**0.002**
SN area by MRI, mm^2^	55.4 ± 7.9	33.1 ± 10.9	**<0.001**
SN area by cTCS, mm^2^	10.8 ± 2.74	24.4 ± 7.49	**<0.001**
Width of 3rd ventricle by MRI, mm	6.19 ± 2.80	6.53 ± 1.98	0.70
Width of 3rd ventricle by cTCS, mm	5.89 ± 1.89	6.58 ± 2.38	0.36

cTCS—conventional transcranial sonography; MRI—magnetic resonance imaging; HC—healthy controls; PD—Parkinson’s disease; RF TCS—radiofrequency transcranial sonography; UPDRS—the Unified Parkinson’s Disease Rating Scale.

**Table 2:** The predictive power of the midbrain tissue displacement signal parameters or size of substantia nigra area for the likelihood that the subject has Parkinson’s disease. Results of logistic regression analyses.

Parameter Estimate	β	Exp(β)	Exp(β) 95% CI	*p* Value
**RF** **TCS 1st Model**				
RMS_exG_SD	−3.490	0.030	[0.001, 0.684]	0.028
FreqHP_Q3	−4.392	0.012	[0.000, 0.857]	0.042
FreqHP_Q3 × RMS_exG_SD	1.806	6.084	[1.352, 27.37]	0.019
Constant	7.235	1387.2	-	0.112
**RF TCS 2nd Model**				
RMS_exG_SD	−2.807	0.060	[0.004, 0.917]	0.043
FreqHP_Q3	−3.627	0.027	[0.001, 1.364]	0.071
FreqHP_Q3 × RMS_exG_SD	1.527	4.606	[1.212, 17.50]	0.025
Age	−0.120	0.887	[0.811, 0.971]	0.010
Constant	12.873	3.898 × 10^5^	–	0.012
**RF TCS 3rd Model**				
RMS_exG_SD	−2.895	0.055	[0.002, 1.433]	0.081
FreqHP_Q3	−3.915	0.020	[0.000, 2.249]	0.104
FreqHP_Q3 × RMS_exG_SD	1.599	4.946	[0.986, 24.806]	0.052
Age	−0.135	0.873	[0.791, 0.964]	0.007
Relative energy in 4–6 Hz	163.25	7.917 × 10^70^	[0.000, 1.083 × 10^180^]	0.203
Constant	13.448	6.923 × 10^5^	–	0.018
**cTCS 1st Model**				
TCS_SN_area	0.731	2.076	[1.319, 3.267]	0.002
Constant	−11.868	0.000	–	0.001
**cTCS 2nd Model**				
TCS_SN_area	0.808	2.243	[1.239, 4.063]	0.008
Age	−0.119	0.888	[0.694, 1.136]	0.345
Constant	−5.084	0.006	–	0.463
**MRI** **1st Model**				
MRI_SN_area	−0.225	0.798	[0.706, 0.902]	<0.001
Constant	9.633	1.526 × 10^4^	–	0.001
**MRI 2nd Model**				
MRI_SN_area	−0.264	0.768	[0.652, 0.905]	0.002
Age	−0.202	0.817	[0.680, 0.981]	0.031
Constant	23.973	2.578 × 10^10^	–	0.006

β—logistic regression coefficient; Exp(β)—odds ratios; CI—confidence interval; RF TCS—radiofrequency transcranial sonography; cTCS—conventional transcranial sonography; MRI—magnetic resonance imaging; FreqHP_Q3—frequency of high-end spectra peak, third quartile, Hz; RMS_exG_SD—root mean square, standard deviation of ex-Gaussian distribution, µm; TCS_SN_area – area of substantia nigra by conventional transcranial sonography, mm^2^; MRI_SN_area—area of substantia nigra by magnetic resonance imaging, mm^2^.

**Table 3:** ROC analysis of the predicted probability of logistic regression models for the likelihood that the subject has Parkinson’s disease.

Model	AUC, %	95% CI	*p* Value	Cut-Off, %	Sensitivity, %	Specificity, %	Overall Correct Classification, %
RF TCS 1st	80.9	[69.1, 92.7]	<0.001	29.9	85.0	69.0	75.5
RF TCS 2nd	86.2	[74.9, 97.6]	<0.001	44.6	80.0	86.2	83.7
RF TCS 3rd	88.3	[78.6, 97.9]	<0.001	42.7	80.0	86.2	83.7
cTCS 1st	98.2	[94.9, 100.0]	<0.001	77.7	90.0	100.0	95.9
cTCS 2nd	98.7	[96.5, 100.0]	<0.001	70.8	90.0	100.0	95.9
MRI 1st	94.1	[86.5, 100.0]	0.039	54.1	90.0	96.6	93.9
MRI 2nd	97.8	[94.3, 100.0]	0.018	43.3	95.0	96.6	95.9

AUC—area under a curve; CI—confidence interval; cTCS—conventional transcranial sonography; MRI—magnetic resonance imaging; RF TCS—radiofrequency transcranial sonography.

## Abbreviations

AUC	area under a curve
FreqD	dominant frequency
FreqHP	frequency of high-end spectra peak
HC	healthy controls
IQR	interquartile range
LR	logistic regression
MRI	magnetic resonance imaging
PD	Parkinson’s disease
RF	radiofrequency
RMS	root mean square
ROI	region of interest
ROC	receiver operating characteristic
RMS	root mean square
SD	standard deviation
SN	substantia nigra
TCS	transcranial sonography
UPDRS	the Unified Parkinson’s disease rating scale
US	ultrasound
